# WeChat as a Platform for Baduanjin Intervention in Patients With Stable Chronic Obstructive Pulmonary Disease in China: Retrospective Randomized Controlled Trial

**DOI:** 10.2196/23548

**Published:** 2021-02-02

**Authors:** Junjie Bi, Wei Yang, Ping Hao, Yongmei Zhao, Dan Wei, Yipeng Sun, Yuhua Lin, Meng Sun, Xuan Chen, Xuming Luo, Shanqun Li, Wei Zhang, Xiongbiao Wang

**Affiliations:** 1 Department of Respiratory Medicine Putuo Hospital Shanghai University of Traditional Chinese Medicine Shanghai China; 2 Department of Nursing Putuo Hospital Shanghai University Of Traditional Chinese Medicine Shanghai China; 3 Department of Respiratory Medicine Shuguang Hospital Shanghai University of Traditional Chinese Medicine Shanghai China; 4 Department of Pulmonary Medicine Zhongshan Hospital Fudan University Shanghai China

**Keywords:** WeChat management, chronic obstructive pulmonary disease, Baduanjin rehabilitation

## Abstract

**Background:**

Pulmonary rehabilitation is a crucial part of the nonpharmacological treatment of stable chronic obstructive pulmonary disease (COPD), but management remains problematic. WeChat could serve as a useful tool in patient management. Baduanjin is a popular exercise in China that is usually applied in pulmonary rehabilitation, which has been confirmed to be effective in improving lung function and life quality.

**Objective:**

This study aimed to explore the efficiency of WeChat in the management of Baduanjin exercise in COPD patients.

**Methods:**

A total of 200 patients from the respiratory department of Putuo Hospital participated in the Baduanjin rehabilitation project from September 2018 to October 2019, and were randomly assigned to the WeChat and control groups and followed up using the WeChat platform or telephone for 12 weeks. The frequency of Baduanjin exercise, lung function (percentage of forced expiratory volume in 1 second predicted, FEV1% predicted), and COPD assessment test (CAT) scores were collected and compared between the two groups. The number of message exchanges and a satisfaction survey on the WeChat platform were used to assess the feasibility of WeChat management outside the hospital.

**Results:**

The Baduanjin exercise frequency significantly differed between the control group and WeChat group (*F*=33.82, *P<*.001) and across various time points (*F*=214.87, *P<*.001). After the follow-up on WeChat, there were fewer patients not performing Baduanjin exercise. The FEV1% predicted value significantly differed before and after Baduanjin exercise in the control group (*Z*=−3.686, *P*<.001) and the WeChat group (*Z*=−6.985, *P<*.001). A significant difference in the FEV1% predicted value was observed after Baduanjin exercise between the two groups (*Z*=−3.679, *P*<.001). The CAT score significantly differed before and after Baduanjin exercise in the control group (*Z*=−4.937, *P<*.001) and the WeChat group (*Z*=−5.246, *P*<.001). A significant difference in the CAT score was observed after Baduanjin exercise between the two groups (*Z*=−5.246, *P<*.001). The number of completed Baduanjin exercises, lung function, and CAT scores in active patients were higher than those in nonactive patients. All satisfaction survey items were scored with more than 4 points. Among the items, the highest score (mean 4.54, SD 0.77) was for continued WeChat management, followed by the effective management of Baduanjin exercise (mean 4.46, SD 0.87). The patients in the WeChat group showed much higher enthusiasm for and compliance with Baduanjin exercise, resulting in better life quality and lung function. The patients were very satisfied with the WeChat management because of the obvious curative effect and home feeling.

**Conclusions:**

The WeChat platform provided a feasible, effective, and sustainable management plan for Baduanjin rehabilitation.

**Trial Registration:**

Chinese Clinical Trial Registry ChiCTR1900028248; http://www.chictr.org.cn/showprojen.aspx?proj=46995

## Introduction

Chronic obstructive pulmonary disease (COPD) is a chronic disease characterized by persistent respiratory symptoms and airflow limitation. COPD develops progressively and is related to an abnormal inflammatory response to harmful gases or particles [1]. COPD, ischemic heart disease, cerebrovascular disease, and cancer are the four major causes of human death. The prevention and treatment of COPD have introduced substantial economic burdens for families and countries [2]. The currently used drugs cannot prevent the progression of COPD. The incidence is approximately 13.6% among persons older than 40 years in China [3], and patients usually have a low quality of life with a high disability rate. As a chronic disease, COPD is prone to acute exacerbation [4]. The goals of COPD treatment are to reduce the symptoms, reduce the frequency and severity of exacerbations, and improve the health status and exercise tolerance [5]. Except for hospitalization during acute exacerbation, most patients need to perform self-care at home for many years. Therefore, the management of stable patients is especially important [6]. However, most COPD patients lack disease-related knowledge. Although medical staff members provide disease health knowledge education using different modes, such as education during hospitalization, owing to differences in patient awareness of the disease, education level, compliance, and other factors, many patients cannot effectively manage themselves [7]. It is difficult to meet the needs of the increasing morbidity of COPD and satisfy the increasing expectation of a quality life with the existing medical and health service model in China. Therefore, it is necessary to explore a new management model for stable COPD patients [8].

Pulmonary rehabilitation for COPD has been given more attention in recent years. Pulmonary rehabilitation has been demonstrated to improve the symptoms of dyspnea, improve exercise endurance, reduce the number of in-hospital days, and reduce the frequency of acute exacerbation [9]. Baduanjin, as a traditional aerobics exercise, is listed as the 97th sports item officially launched by the State General Administration of Sport in 2003 and has been widely promoted in China. Baduanjin involves the following eight components: (1) pushing up the heavens, (2) drawing the bow, (3) separating heaven and earth, (4) shaking the heavenly pillar, (5) punching with an angry gaze, (6) shaking the head and swaying the buttocks to extinguish fire in the heart, (7) touching the toes and bending back, and (8) bouncing on the toes. Baduanjin has been applied in lung rehabilitation for many years in China. Previous studies have confirmed that Baduanjin has a strong clinical effect on lung rehabilitation in COPD patients [10]. Thus, Baduanjin has become an exercise prescription for COPD [11]. However, owing to the lack of effective management, pulmonary rehabilitation, including Baduanjin, has not been practiced widely and continuously, and the benefit for patients is limited. In addition, there is a lack of consistency in the length of the research period among many published studies [12].

With the key position of home-based physical activity, long-term management becomes critical. With the rapid development of computer technology, network technology, and multimedia technology, new media have become convenient, fast, interactive, and wide-spread platforms for information dissemination. WeChat is the mainstream instant communication platform in China [13]. The “2018 WeChat Annual Data Report” reported that 1.01 billion users logged on to WeChat daily in 2018. The function of the platform is quite strong but is very easy to operate. Various message forms, such as text, picture, voice, and video, could be presented in communication. WeChat has rapidly developed into a comprehensive information platform integrating communication, information, entertainment, search, e-commerce, office collaboration, and corporate customer service. It is reasonable to apply new technology in medical areas. The “Internet Medical” model is very suitable for China’s current situation, that is, a large population with a shortage of medical resources [14]. WeChat has been gradually used in medical education and the follow-up of patients, and it has produced successful outcomes [15,16].

Because the benefits disappear over time if activity and other good behaviors are not continued, COPD patients should be offered consistent guidance. The telephone has been used for follow-up in early studies but can no longer meet the requirements [17]. New technologies, such as WeChat, could be applied for more effective management. Thus, we established a new system for continuous management from inside the hospital to outside the hospital under the WeChat platform. In the hospital, patients are provided disease knowledge and educated regarding the skills of pulmonary rehabilitation, such as Baduanjin, diet nutrition, correct drug use, etc [18,19]. After discharge, all patients are placed under management using the WeChat platform.

As Baduanjin requires long-term persistence by COPD patients, consistent encouragement from medical staff is essential. In this study, the established WeChat platform was applied to manage Baduanjin exercise in patients with stable COPD, and the results are encouraging.

## Methods

### Research Design and Flow Chart

**Figure 1:**
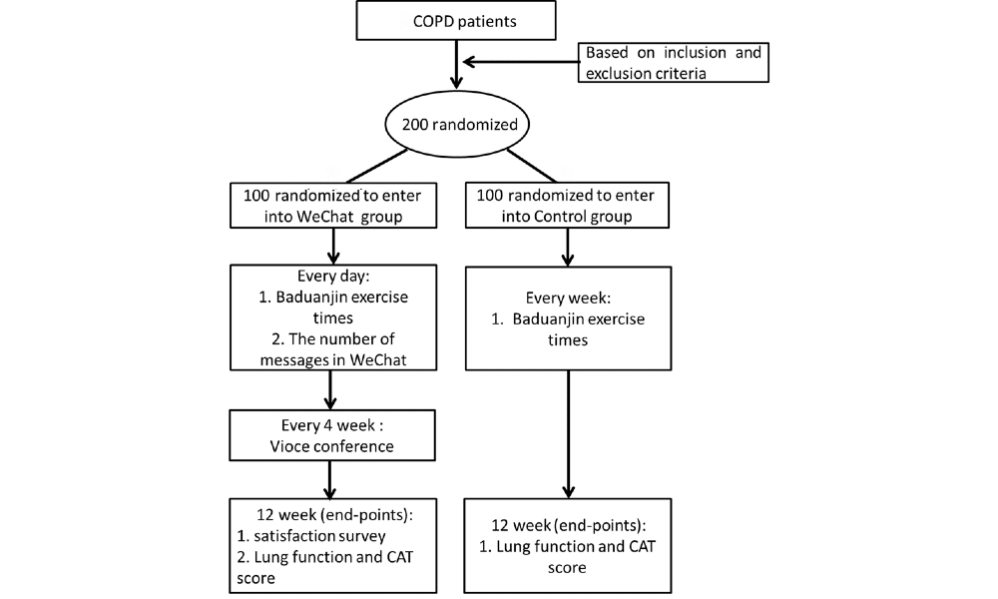
Flowchart of the research design. CAT: chronic obstructive pulmonary disease assessment test; COPD: chronic obstructive pulmonary disease.

This study was a parallel controlled study conducted from September 2018 to October 2019 in Shanghai, China. Two hundred stable COPD patients were included, and the participants were randomly divided into the following two groups (1:1 ratio): the WeChat intervention group and routine nursing control group (Figure 1). The research plan was approved by the ethics committee of Putuo Hospital (grant number: PTEC-A-2018-25-1).

### Procedures of Baduanjin Exercise

The rehabilitation therapist instructed the patients to perform Baduanjin exercise until they could accomplish proficiency according to the Baduanjin video (produced by the State Sports General Administration in 2003). All patients were asked to perform the exercise two to four times a day for no less than 5 days per week.

### Establishment of the WeChat Platform

A WeChat platform team with six members was established. The head nurse of the ward served as the team leader and was responsible for the operation and guidance of the entire project. Two nurse supervisors were responsible for teaching the patients and their families how to use the platform, providing all educational documents to the patients at the appropriate time, maintaining contact with the patients, and urging the patients to perform Baduanjin exercise. Two physicians were responsible for evaluating the patients’ condition and devising the medical plan. One technician in the pulmonary function room was responsible for evaluating the lung function of all patients, collecting the data, and performing statistical analyses of the data. The team members cooperated very well, and the platform was operated effectively. The patients very actively participated in the communication. Responses to all questions were obtained from the medical staff in a timely manner.

### Participants

All patients with stable COPD were discharged from the respiratory medicine ward of a large general hospital. All candidates first completed a brief screening questionnaire. Patients who met the inclusion criteria were invited to participate in the study and received more detailed information regarding the study. After providing written informed consent, all qualified patients were divided into the WeChat group and control group.

The inclusion criteria were as follows: (1) 50 to 80 years of age regardless of gender; (2) confirmed clinical diagnosis of stable COPD according to the standard of GOLD 2018 [20]; (3) informed consent (patients and families); (4) ability to use WeChat proficiently (patients and primary caregivers); and (5) ability to perform Baduanjin independently.

The exclusion criteria were as follows: (1) severe heart, liver, and kidney diseases, tumors, or other conditions that may affect the observation; (2) life expectancy less than 1 year; and (3) history of conducting physical exercise for a long time (≥3 times/week, ≥20 minutes/time, persisting for more than 12 months) [21].

Pulmonary function (percentage forced expiratory volume in 1 second [FEV1%]) was the outcome index. Based on previous studies showing FEV1% improvement in clinical trials [22], the mean and SD of FEV1% in the control group were 57.09 and 22.53, respectively, while the mean and SD of FEV1% in the experimental group were 60.24 and 20.15, respectively. The alpha level was set to .05, the power was 80%, and the participant dropout rate was 20%. A sample size of 192 patients (96 per group) was required for the primary analysis. Thus, we recruited 200 patients in the trial (100 in the WeChat group and 100 in the control group).

### Randomization and Masking

Using the method of block randomization, 200 research patients were randomly assigned to the WeChat and control groups. The block size was defined as four, and there were six sequential arrangements and combinations. Excel (Microsoft Corp) randomly generated 50 (1‒50) numbers that did not repeat. Then, the numbers were divided by six to obtain the remainder, and six combinations were matched according to the remainder. A random block group table was then completed. After the research patients were included, the random block table was assigned to a group. By the nature of the trial design, neither the research staff nor the participants were blinded to the intervention.

### Intervention Method

#### Control Group

Handbooks were distributed to the participants, and they included the following: (1) a Baduanjin video (produced by the State Sports General Administration in 2003); (2) Baduanjin notes; (3) information regarding COPD disease; (4) the importance of pulmonary rehabilitation; (5) nutrition and diet suggestions; and (6) prevention of acute exacerbation of COPD. The patients were asked to comply with medical advice, including performing Baduanjin exercise at home and being followed up by telephone once a week. The chronic obstructive pulmonary disease assessment test (CAT) questionnaire and lung function evaluation were performed before and after the study using a spirometer.

#### WeChat Group

The participants also received the necessary education on WeChat. All education materials, including text, pictures, videos, and voice messages, were sent to the patients as an electric document via WeChat and individuals as needed. The patients were supervised while performing Baduanjin and maintaining healthy behaviors.

A WeChat voice conference was held every 4 weeks for 40 minutes. The medical staff were required to be online, and they held a seminar regarding common problems that the patients usually discussed and answered questions.

The feedback information was as follows: (1) dyspnea status; (2) discomfort status; (3) appetite status; (4) number of Baduanjin exercises completed every day (the patients in the WeChat group submitted their information online every day); (5) completion of the satisfaction survey by all patients at the end of the 12th week; and (6) the CAT questionnaire and lung function evaluation before and after the study.

### Quality Control

A designated attending physician supervised the project every month and evaluated the progress.

### Outcome Measures

#### Baseline Assessment

The general demographic data included the patients’ gender, age, smoking status, disease course, lung function, classification of airflow limitation severity, comorbidities, and combined COPD assessment according to GOLD 2018.

#### Data Collection and Evaluation at the Endpoint

The Baduanjin exercise frequency was collected each week in WeChat and once a week by telephone. At the end of the study, the lung function evaluation, quality of life evaluation (CAT), personal activity evaluation, and satisfaction survey were completed by all participants.

##### Lung Function and CAT

All patients underwent a spirometric analysis before and after the trial. Given the noninvasive and simple characteristics of lung function tests, they are generally adopted in clinical practice for early diagnosis and prognostic evaluations. Lung function tests can effectively reflect the pathological changes in the airway and their degree, and provide reliable results of the presence and severity of spirometric abnormalities. The CAT score is a reliable and effective indicator used to assess the living conditions and quality of life of COPD patients. The CAT questionnaire is very simple, can be completed by patients in 2 to 3 minutes, and includes eight items, including daily living ability and physical health. Compared with the St. George’s Respiratory Questionnaire (SGRQ), the content is greatly reduced, but the required indicators are comprehensive and can more intuitively reflect changes in patient health. Related studies have shown that the lung function of patients with COPD is correlated with the CAT score [23] and that the CAT score has good repeatability [24]. Thus, the CAT score is a good evaluation of stable COPD.

##### Activation of the Platform

We recorded the number of messages exchanged on the WeChat platform. The communication included text/graphics, voice, pictures, videos, etc. The activity on the platform and the degree of concern regarding the disease were evaluated.

##### Assessment of Satisfaction With the WeChat Platform Management

A self-designed questionnaire was used to evaluate the satisfaction of the participants at the end of the trial. In total, the following eight items were assessed: effective management of Baduanjin exercise, practical information, convenient communication and interaction, smooth platform operation, simple operation process, continued WeChat management, effective COPD rehabilitation management, and service attitude of the medical staff. A 5-point Likert scale was used with scores ranging from 1 to 5 points (1, strongly disagree; 2, disagree; 3, uncertain; 4, agree; and 5, strongly agree) [25]. A higher score was associated with better COPD patient experience and service evaluation of the WeChat platform and better effectiveness of the WeChat management of Baduanjin exercise.

### Statistical Analysis

The baseline data were based on continuous measurement data and categorized count data. The measurement data are expressed as mean (SD) or median (IQR). The count data are characterized as frequencies. Two-tailed *t* tests (mean and SD) or chi-squared tests were used to analyze the normally distributed data. A nonparametric test was used to analyze the nonnormally distributed data (median and IQR).

To analyze the results, repeated measures analysis of variance, a simple effect model, and two-sample *t* tests were used. To determine the within-group differences and between-group differences, the 95% CIs were calculated for the continuous measures. The statistical analysis was performed using SPSS 22.0 software (IBM Corp), and *P*<.05 was considered statistically significant.

## Results

### Sample Characteristics

Based on the inclusion and exclusion criteria, we screened 200 patients and completed baseline assessments before randomization to the trial of 100 patients in each group. Of the 200 patients, 67 were female and 133 were male. The ratio of male and female patients assigned to the two groups was not different. The mean age was 68.16 years (WeChat group, 69.43 years; control group, 68.87 years), and the mean course of COPD was 4.46 years (WeChat group, 4.50 years; control group, 4.31 years). Most patients had severe COPD, accounting for 73% (146/200) of cases (WeChat group, 72% [72/100]; control group, 74% [74/100]). Patients with moderate and very severe COPD accounted for 11.5% (23/200) of cases (WeChat group, 12% [12/100]; control group, 11% [11/100]) and 15.5% (31/200) of cases (WeChat group, 16% [16/100]; control group, 15% [15/100]), respectively. We also assessed the number of acute exacerbations and current smokers in both groups. Patients who had acute exacerbation at least once in the past year accounted for 31% (31/100) of cases in the control group and 35% (35/100) of cases in the WeChat group. The proportion of current smokers in the control group (34% [34/100]) was comparable to that in the WeChat group (37% [37/100]). According to statistical analysis, the baseline characteristics were balanced in both groups (Table 1).

**Table 1:** Baseline characteristics of the participants in the WeChat and control groups.

Baseline characteristics		Control group (n=100)	WeChat group (n=100)	*P* value
Gender (female/male), n		32/68	35/65	.76
Age (years), mean (SD)		68.87 (5.33)	69.43 (7.38)	.54
Course (years), mean (SD)		4.31 (2.07)	4.50 (2.46)	.56
Acute exacerbation^a^, n (%)		31 (31%)	35 (35%)	.65
Current smoker, n (%)		34 (34%)	37 (37%)	.77
**Severity, n**				.95
	Moderate	11	12	
	Severe	74	72	
	Very severe	15	16	

^a^Acute exacerbation: at least once in the past year.

### Comparison of the Baduanjin Exercise Frequency Between the Control and WeChat Groups

**Figure 2:**
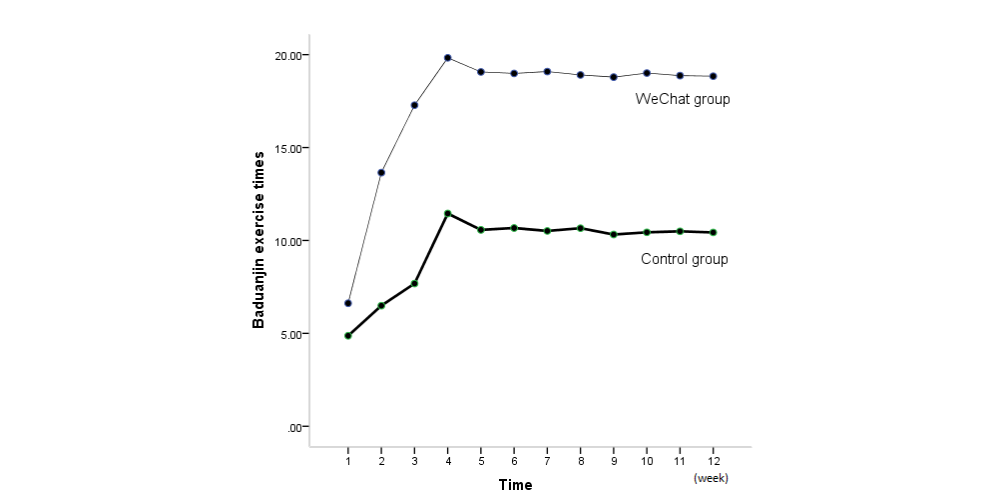
Comparison of Baduanjin exercise frequency per week (mean) between the WeChat group and control group (all *P*<.001).

A repeated measures analysis of variance was performed to analyze the data from the two groups. The data did not conform to the sphericity test (*P*<.001). The analysis of the multivariate test revealed that in the within-group comparisons in both the control and WeChat groups, the Baduanjin exercise frequency significantly differed across various time points (*F*=214.87, *df*=11, *P*<.001). This finding indicates that in both the control and WeChat groups, Baduanjin management was effective. Additionally, in the between-group comparisons, the different groups (control group vs WeChat group) showed a significant difference in the time points (*F*=33.82, *df*=1, *P*<.001) (Multimedia Appendix 1, Figure 2).

Further analysis by a simple effects model showed a difference within and between the groups. The Baduanjin exercise frequencies in the control and WeChat groups (1 week to 4 weeks) increased from week 1 to week 4 and then maintained a plateau until the end of the project. In the within-group comparisons, there were statistically significant differences at each time point in weeks 1 to 3 compared with week 4, but no difference was observed between weeks 5 and 12 and week 4. According to the between-group comparison results, significant differences were found between the WeChat and control groups at the same time points (all *P*<.001). We found that WeChat management was more effective for performing Baduanjin exercise (Multimedia Appendix 1, Figure 2).

### Lung Function Assessment

**Figure 3:**
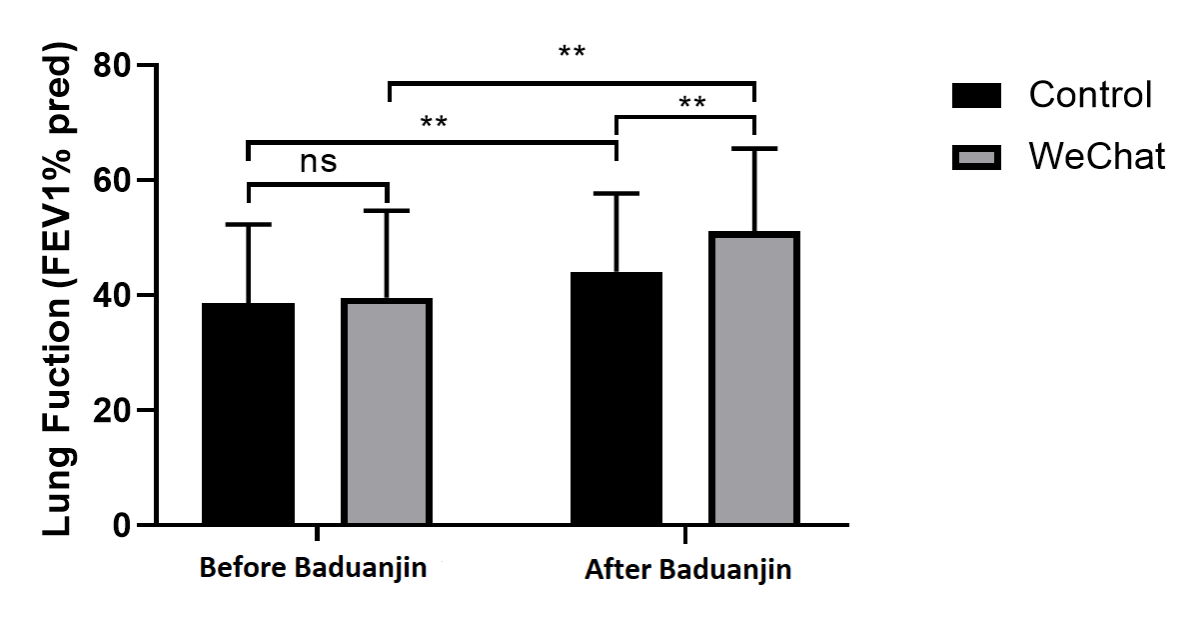
Lung function before and after Baduanjin exercise. FEV1% pred: percentage of forced expiratory volume in 1 second predicted; ns: not significant. **P<.001.

The FEV1% predicted value significantly differed before and after the trial in both the control group (*Z*=−3.686, *P*<.001) and WeChat group (*Z*=−6.985, *P*<.001), and the FEV1% predicted value in both groups improved after Baduanjin exercise. A significant difference was found in the FEV1% predicted values after Baduanjin exercise between the two groups (*Z*=−3.679, *P*<.001), and no difference was observed before the trial (Multimedia Appendix 2, Figure 3). The results demonstrate that Baduanjin exercise was more effective in the WeChat group.

### CAT Score Evaluation

**Figure 4:**
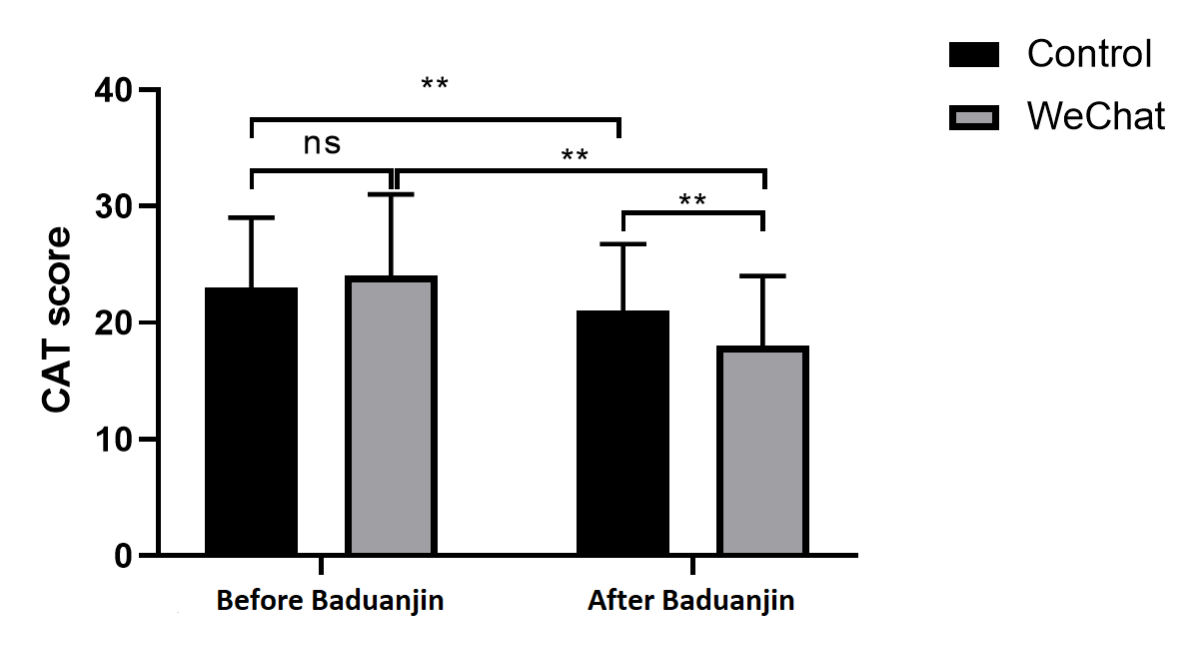
Chronic obstructive pulmonary disease assessment test (CAT) score before and after Baduanjin exercise. ns: not significant. **P<.001.

All patients completed the questionnaire independently. We found that the CAT score significantly differed before and after the exercise in the control group (*Z*=−4.937, *P*<.001) and the WeChat group (*Z*=−5.246, *P*<.001). The life quality of the patients in both groups improved after Baduanjin exercise (Figure 4). No statistically significant difference was found in the CAT score between the two groups before the study (*Z*=−1.407, *P*=.30), and a significant difference was found after the exercise (*Z*=−5.246, *P*<.001). These results demonstrate greater improvement in the life quality of patients in the WeChat group after the exercise (Multimedia Appendix 3, Figure 4).

### Activity and Interaction on the WeChat Platform

Communication in the WeChat group was mainly conducted via text/graphics, with 13,911 items, followed by voice messages, with a total of 1317 items. Text/graphics and voice accounted for the main methods of message exchange. Pictures and videos were also used; however, the frequency was relatively low (Table 2).

**Table 2:** Distribution of different message forms present on the WeChat platform.

Message form	1-4 weeks, n	5-8 weeks, n	9-12 weeks, n	Total (N=16,100), n (%)
Text/graphics	4680	4601	4630	13,911 (86.40%)
Voice	388	428	501	1317 (8.18%)
Picture	173	200	289	662 (4.11%)
Video	52	54	77	183 (1.14%)
Other files	10	7	10	27 (0.17%)

All messages could be divided into the following three groups: information provided by the medical team, medical staff-patient interaction communication, and patient-patient interaction communication. The frequency of the provided information was relatively fixed. Medical staff-patient interaction was relatively frequent during the first 2 weeks but was less frequent thereafter. However, patient-patient interaction gradually increased, indicating that a COPD family was established. Interestingly, we found that the patients interacted and encouraged each other. The platform became a common home for all WeChat participants.

**Figure 5:**
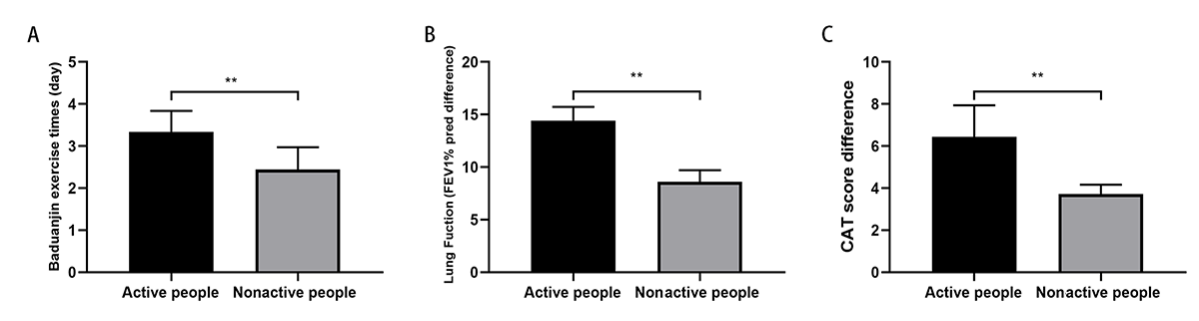
Comparison of outcomes between active patients and nonactive patients. (A) Daily exercise frequency; (B) Difference in lung function before and after Baduanjin exercise; and (C) Difference in the chronic obstructive pulmonary disease assessment test (CAT) score before and after Baduanjin exercise. FEV1% pred: percentage of forced expiratory volume in 1 second predicted.**P<.001.

We divided the WeChat group into active patients and nonactive patients based on the number of messages exchanged. Those with more than two messages per day were considered active patients, and those with one or fewer messages were considered inactive patients. Eighty-six patients were more active in the group. The number of completed Baduanjin exercises, lung function, and CAT scores of these active patients were higher than those of the nonactive patients (Figure 5). The amount of information exchanged by the WeChat group reflected the activity of the WeChat management platform, indicating that COPD patients actively participated in the management of the disease and received better outcomes.

### Evaluation of the Satisfaction With the WeChat Platform

According to the score of the 5-point Likert scale, items that received more than 4 points were as follows: continued WeChat management, effective Baduanjin exercise management, practical information content, convenient communication and interaction, smooth platform operation, simple operation process, service attitude of the medical staff, and COPD rehabilitation. The results indicated that the COPD patients were satisfied with the management of the WeChat platform. Among these items, the highest score was for continued WeChat management (mean 4.54, SD 0.77), followed by effective management of Baduanjin exercise (mean 4.46, SD 0.87), indicating the feasibility, sustainability, and effectiveness of disease management with the WeChat platform (Table 3).

**Table 3:** Satisfaction scores of the WeChat platform.

Contents	Agreement (N=50)^a^					Score, mean (SD)
	Strongly agree, n (%)	Agree, n (%)	Uncertain, n (%)	Disagree, n (%)	Strongly disagree, n (%)	
Continued WeChat management	28 (56%)	21 (42%)	1 (2%)	0 (0%)	0 (0%)	4.54 (0.77)
Effective Baduanjin exercise management	26 (52%)	21 (42%)	3 (6%)	0 (0%)	0 (0%)	4.46 (0.87)
Practical information content	22 (44%)	28 (56%)	0 (0%)	0 (0%)	0 (0%)	4.44 (0.71)
Convenient communication and interaction	24 (48%)	23 (46%)	3 (6%)	0 (0%)	0 (0%)	4.42 (0.86)
Smooth platform operation	21 (42%)	28 (56%)	1 (2%)	0 (0%)	0 (0%)	4.40 (0.76)
Simple operation process	21 (42%)	28 (56%)	1 (2%)	0 (0%)	0 (0%)	4.40 (0.76)
Service attitude of the medical staff	24 (48%)	20 (40%)	6 (12%)	0 (0%)	0 (0%)	4.36 (0.98)
COPD^b^ rehabilitation	22 (44%)	22 (44%)	6 (12%)	0 (0%)	0 (0%)	4.30 (0.97)

^a^Strongly agree, 5 points; agree, 4 points; uncertain, 3 points; disagree, 2 points; strongly disagree, 1 point.

^b^COPD: chronic obstructive pulmonary disease.

## Discussion

### Principal Findings

**Figure 6:**
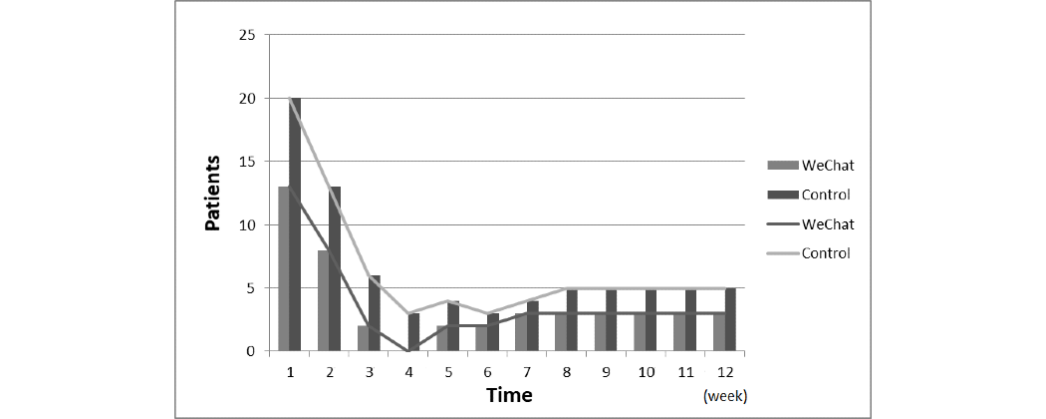
Number of patients with a Baduanjin exercise frequency of 0 (per week) in the WeChat group and control group.

The main purpose of this study was to test the feasibility and efficiency of WeChat in the management of Baduanjin exercise in COPD patients. The results of this study suggest that the management of Baduanjin exercise with the WeChat platform improves the enthusiasm and compliance of patients. The frequency of Baduanjin exercise using the WeChat-based intervention was 0 to 21 times (mean 18.84) to 0 to 13 times (mean 10.43) per person per week compared with traditional interventions at 12 weeks. The Baduanjin exercise frequency in the WeChat group was markedly higher than that in the control group. Under the management of WeChat, some patients did not perform Baduanjin exercise. However, after the follow-up on WeChat, the number of patients with a Baduanjin exercise frequency of 0 decreased (Figure 6). Therefore, Baduanjin exercise management based on the WeChat platform improved the enthusiasm and compliance of patients. These results are consistent with the beneficial effect of compliance using WeChat management in other clinical populations [26].

**Figure 7:**
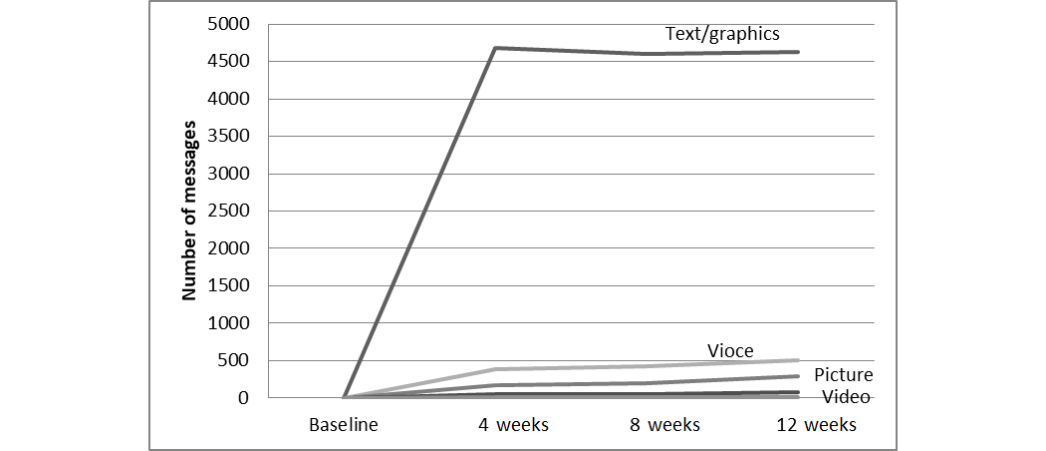
Trend of messages exchanged on WeChat.

Based on the WeChat platform, communication between medical staff and patients increased, and the effectiveness of Baduanjin lung rehabilitation improved. Studies have shown that the frequency of the use of the platform is crucial for the effectiveness of the intervention [27]. Our results indicate that the total number of messages exchanged gradually increased and reached a peak at approximately 4 weeks; thereafter, the number maintained a plateau. Interaction with text/graphics exhibited the most obvious increase (Figure 7). We also found that active participants had better outcomes. The interaction between patients increased, and mutual exchange, sharing, and supervision promoted more enthusiasm among the patients. The role of the team was always favorable.

All participants completed the satisfaction survey, and a high score was obtained in the WeChat group. Based on the questionnaire responses, we found that continuing WeChat management and Baduanjin exercise management were the top two ranked items. The other items also achieved relatively high scores. Thus, the patients expected to continue WeChat management, and the WeChat management of Baduanjin exercise is feasible.

Early hospital follow-up, coaching, COPD action plans and management programs, telemedicine, pulmonary rehabilitation, and other interventions require more research. Improvement in management is an effective strategy for improving patient recovery [28]. A recent study also noted that reducing readmission could improve prognosis [29]. Although the management of COPD patients has been studied since the 1960s [30], the situation of management is not optimistic. Many problems are associated with COPD management, and a key problem is lung rehabilitation [31]. Currently, published data lack sufficient evidence for the identification of an effective pathway to improve the symptoms of COPD by management. Therefore, better ways to achieve management must be explored [32].

With the development of the internet, major communication platforms have begun to be applied in health care. Social media platforms, such as Twitter and Facebook, have been steadily applied in medical education [33,34]. Facebook has been used to study many aspects of health care, and it provides a convenient and easily accessible way to collect unsolicited and observational patient data [35]. An analysis of the content of COPD group chats on Facebook showed that COPD Facebook group members share specific disease-related experiences and request information regarding select self-management topics. This information can be used to improve the quality of self-management support provided to members of popular COPD Facebook groups [36]. WeChat is also gradually being used in medical teaching and the clinic, but the clinical application needs improvement [37]. Clinical studies based on the WeChat platform, such as cough variant asthma and promoting weight loss, have achieved good results [38]. The internet could also help patients with COPD understand the disease and manage the condition by themselves [39].

### Limitations

This study has some limitations. First, the study period was quite short (approximately 12 weeks), which may have affected the judgement of compliance, but our study is ongoing, and more data will be reported in the future. Second, the number of participants in this study was relatively small, although the sample size was calculated statistically. However, because of the complexity of clinical trials and the uncontrollability of patients, a larger number of patients could supply more intact information. Third, as the original data were obtained from patient reports, inaccurate reports could not be fully excluded. Thus, there is a problem of credibility in patient reports using WeChat management. Finally, our study only explored the effectiveness of Baduanjin rehabilitation training under WeChat platform management. More projects in self-management supported by the WeChat platform could be fully studied in the future.

### Conclusions

In this study, a new management platform involving WeChat from the in-hospital period to the out-of-hospital period was established for patients. Pulmonary rehabilitation using Baduanjin exercise for COPD patients was applied using this platform. This online rehabilitation training model met the patients’ requirements as this platform was convenient, easy to understand, and time saving. Information is shared in a timely manner, and seamless communication is maintained with patients. Additionally, the compliance and enthusiasm of the patients were much higher. Baduanjin exercise based on WeChat platform management was effective in improving the life quality and lung function of COPD patients. Patient management based on WeChat is effective, feasible, and sustainable.

## Appendix

Multimedia Appendix 1 Effects of the WeChat intervention on Baduanjin exercise times.

Multimedia Appendix 2 Lung function in the WeChat group and control group.

Multimedia Appendix 3 Chronic obstructive pulmonary disease assessment test score in the WeChat group and control group.

Multimedia Appendix 4 CONSORT-eHEALTH checklist.

## Abbreviations

CAT: chronic obstructive pulmonary disease assessment test
COPD: chronic obstructive pulmonary disease
FEV1%: percentage of forced expiratory volume in 1 second

## References

[1] Dobler CC, Morrow AS, Beuschel B, Farah MH, Majzoub AM, Wilson ME, Hasan B, Seisa MO, Daraz L, Prokop LJ, Murad MH, Wang Z (2020). Pharmacologic Therapies in Patients With Exacerbation of Chronic Obstructive Pulmonary Disease. Ann Intern Med.

[2] Zhu B, Wang Y, Ming J, Chen W, Zhang L (2018). Disease burden of COPD in China: a systematic review. Int J Chron Obstruct Pulmon Dis.

[3] Fang L, Gao P, Bao H, Tang X, Wang B, Feng Y, Cong S, Juan J, Fan J, Lu K, Wang N, Hu Y, Wang L (2018). Chronic obstructive pulmonary disease in China: a nationwide prevalence study. The Lancet Respiratory Medicine.

[4] Wedzicha JA, Singh R, Mackay AJ (2014). Acute COPD exacerbations. Clin Chest Med.

[5] Vogelmeier CF, Román-Rodríguez M, Singh D, Han MK, Rodríguez-Roisin R, Ferguson GT (2020). Goals of COPD treatment: Focus on symptoms and exacerbations. Respir Med.

[6] Mitchell KE, Johnson-Warrington V, Apps LD, Bankart J, Sewell L, Williams JE, Rees K, Jolly K, Steiner M, Morgan M, Singh SJ (2014). A self-management programme for COPD: a randomised controlled trial. Eur Respir J.

[7] Siltanen H, Aine T, Huhtala H, Kaunonen M, Vasankari T, Paavilainen E (2020). Psychosocial issues need more attention in COPD self-management education. Scand J Prim Health Care.

[8] Sunde S, Walstad RA, Bentsen SB, Lunde SJ, Wangen EM, Rustøen T, Henriksen AH (2014). The development of an integrated care model for patients with severe or very severe chronic obstructive pulmonary disease (COPD): the COPD-Home model. Scand J Caring Sci.

[9] Ain QU, Majeed N (2020). Commentary on: Efficacy of pulmonary rehabilitation in improving the quality of life for patients with chronic obstructive pulmonary disease: Evidence based on nineteen randomized controlled trials. Int J Surg.

[10] Yang Y, Chen K, Tang W, Xie X, Xiao W, Xiao J, Luo X, Wang W (2020). Influence of Baduanjin on lung function, exercise capacity, and quality of life in patients with mild chronic obstructive pulmonary disease. Medicine (Baltimore).

[11] Liu S, Ren Z, Wang L, Wei G, Zou L (2018). Mind⁻Body (Baduanjin) Exercise Prescription for Chronic Obstructive Pulmonary Disease: A Systematic Review with Meta-Analysis. Int J Environ Res Public Health.

[12] McNaughton A, Levack W, McNaughton H (2020). Taking Charge: A Proposed Psychological Intervention to Improve Pulmonary Rehabilitation Outcomes for People with COPD. Int J Chron Obstruct Pulmon Dis.

[13] Chen J, Ho E, Jiang Y, Whittaker R, Yang T, Bullen C (2020). Mobile Social Network-Based Smoking Cessation Intervention for Chinese Male Smokers: Pilot Randomized Controlled Trial. JMIR Mhealth Uhealth.

[14] Cui F, Ma Q, He X, Zhai Y, Zhao J, Chen B, Sun D, Shi J, Cao M, Wang Z (2020). Implementation and Application of Telemedicine in China: Cross-Sectional Study. JMIR Mhealth Uhealth.

[15] Lyu K, Zhao J, Wang B, Xiong G, Yang W, Liu Q, Zhu X, Sun W, Jiang A, Wen W, Lei W (2016). Smartphone Application WeChat for Clinical Follow-up of Discharged Patients with Head and Neck Tumors: A Randomized Controlled Trial. Chin Med J (Engl).

[16] Zhang W, Li ZR, Li Z (2019). WeChat as a Platform for Problem-Based Learning in a Dental Practical Clerkship: Feasibility Study. J Med Internet Res.

[17] Davies C, Vas P, Oyibo SO (2013). Telephone follow-up for the management of thyrotoxicosis: a patient satisfaction survey. J Telemed Telecare.

[18] Kong K, Zhang L, Huang L, Tao Y (2017). Validity and practicability of smartphone-based photographic food records for estimating energy and nutrient intake. Asia Pac J Clin Nutr.

[19] Feng S, Liang Z, Zhang R, Liao W, Chen Y, Fan Y, Li H (2017). Effects of mobile phone WeChat services improve adherence to corticosteroid nasal spray treatment for chronic rhinosinusitis after functional endoscopic sinus surgery: a 3-month follow-up study. Eur Arch Otorhinolaryngol.

[20] Mirza S, Clay RD, Koslow MA, Scanlon PD (2018). COPD Guidelines: A Review of the 2018 GOLD Report. Mayo Clin Proc.

[21] Dasso NA (2019). How is exercise different from physical activity? A concept analysis. Nurs Forum.

[22] Zhang Y, Hu R, Han M, Lai B, Liang S, Chen B, Robinson N, Chen K, Liu J (2020). Evidence Base of Clinical Studies on Qi Gong: A Bibliometric Analysis. Complement Ther Med.

[23] Pothirat C, Chaiwong W, Limsukon A, Deesomchok A, Liwsrisakun C, Bumroongkit C, Theerakittikul T, Phetsuk N (2015). Detection of acute deterioration in health status visit among COPD patients by monitoring COPD assessment test score. Int J Chron Obstruct Pulmon Dis.

[24] Jones PW, Brusselle G, Dal Negro RW, Ferrer M, Kardos P, Levy ML, Perez T, Soler Cataluña J J, van der Molen T, Adamek L, Banik N (2011). Properties of the COPD assessment test in a cross-sectional European study. Eur Respir J.

[25] Kim DY, Kwon H, Nam K, Lee Y, Kwon H, Chung YS (2020). Remote Management of Poststroke Patients With a Smartphone-Based Management System Integrated in Clinical Care: Prospective, Nonrandomized, Interventional Study. J Med Internet Res.

[26] Cao Y, Lin S, Zhu D, Xu F, Chen Z, Shen H, Li W (2018). WeChat Public Account Use Improves Clinical Control of Cough-Variant Asthma: A Randomized Controlled Trial. Med Sci Monit.

[27] Liu W, Wang C, Lin H, Lin S, Lee K, Lo Y, Hung S, Chang Y, Chung KF, Kuo H (2008). Efficacy of a cell phone-based exercise programme for COPD. Eur Respir J.

[28] Hosseini HM, Pai DR, Ofak DR (2019). COPD: Does Inpatient Education Impact Hospital Costs and Length of Stay?. Hosp Top.

[29] Freedman N (2019). Reducing COPD Readmissions: Strategies for the Pulmonologist to Improve Outcomes. Chest.

[30] Hanlon P, Daines L, Campbell C, McKinstry B, Weller D, Pinnock H (2017). Telehealth Interventions to Support Self-Management of Long-Term Conditions: A Systematic Metareview of Diabetes, Heart Failure, Asthma, Chronic Obstructive Pulmonary Disease, and Cancer. J Med Internet Res.

[31] Augustin IML, Spruit MA, Franssen FME, Gaffron S, van Merode F, Wouters EFM (2020). Incorporating Comprehensive Assessment Parameters to Better Characterize and Plan Rehabilitation for Persons with Chronic Obstructive Pulmonary Disease. J Am Med Dir Assoc.

[32] Raymond B, Luckett T, Johnson M, Hutchinson A, Lovell M, Phillips J (2019). Low-intensity educational interventions supporting self-management to improve outcomes related to chronic breathlessness: a systematic review. NPJ Prim Care Respir Med.

[33] Admon AJ, Kaul V, Cribbs SK, Guzman E, Jimenez O, Richards JB (2020). Twelve tips for developing and implementing a medical education Twitter chat. Med Teach.

[34] Junhasavasdikul D, Srisangkaew S, Sukhato K, Dellow A (2017). Cartoons on Facebook: a novel medical education tool. Med Educ.

[35] Egan KG, Israel JS, Ghasemzadeh R, Afifi AM (2016). Evaluation of Migraine Surgery Outcomes through Social Media. Plast Reconstr Surg Glob Open.

[36] Apperson A, Stellefson M, Paige SR, Chaney BH, Chaney JD, Wang MQ, Mohan A (2019). Facebook Groups on Chronic Obstructive Pulmonary Disease: Social Media Content Analysis. Int J Environ Res Public Health.

[37] Dong Y, Wang P, Dai Z, Liu K, Jin Y, Li A, Wang S, Zheng J (2018). Increased self-care activities and glycemic control rate in relation to health education via Wechat among diabetes patients: A randomized clinical trial. Medicine (Baltimore).

[38] He C, Wu S, Zhao Y, Li Z, Zhang Y, Le J, Wang L, Wan S, Li C, Li Y, Sun X (2017). Social Media-Promoted Weight Loss Among an Occupational Population: Cohort Study Using a WeChat Mobile Phone App-Based Campaign. J Med Internet Res.

[39] Paige SR, Stellefson M, Krieger JL, Alber JM (2019). Computer-Mediated Experiences of Patients with Chronic Obstructive Pulmonary Disease. Am J Health Educ.

