# Supplementary material for: WeChat as a Platform for Baduanjin Intervention in Patients With Stable Chronic Obstructive Pulmonary Disease in China: Retrospective Randomized Controlled Trial
Source: JMIR Mhealth Uhealth. 2021 Feb 2;9(2):e23548. doi: 10.2196/23548 (PMC7886617; doi:10.2196/23548)
Supplement: Multimedia Appendix 1 [file mhealth_v9i2e23548_app1.docx]

**Multimedia Appendix 1.** Effects of the WeChat intervention on Baduanjin exercise times

| Follow-up WeChat group(N=100) Control group（N=100） *P-*value  time （95%CI） （95%CI） Group^a^ Time^b^ Group×Time |
| --- |
| 1w 6.62(6.05 to 7.19) 4.87(4.30 to 5.44) ＜.001 ＜.001  2w 13.65(12.91 to 14.39) 6.49(5.75 to 7.23) ＜.001 ＜.001  3w 17.28(16.80 to 17.76) 7.68(7.20 to 8.16) ＜.001 ＜.001  4w 19.83(19.42 to 20.25) 11.45(11.04 to 11.87) ＜.001 —  5w 19.07(18.53 to 19.62) 10.57(10.03 to 11.12) ＜.001 ＞.05  6w 18.99(18.47 to 19.51) 10.67(10.15 to 11.19) ＜.001 ＞.05 ＜.001  7w 19.09(18.49 to 19.69) 10.51(9.91 to 11.11) ＜.001 ＞.05  8w 18.91(18.28 to 19.54) 10.66(10.03 to 11.29) ＜.001 ＞.05  9w 18.79(18.17 to 19.41) 10.32(9.70 to 10.94) ＜.001 ＞.05  10w 19.01(18.36 to 19.64) 10.44(9.81 to 11.07) ＜.001 ＞.05  11w 18.87(18.24 to 19.50) 10.49(9.86 to 11.12) ＜.001 ＞.05  12w 18.84(18.21 to 19.47) 10.43(9.80 to 11.06) ＜.001 ＞.05 |

^a^Indicates mean change between control group and WeChat group.

^b^Indicates mean change with follow-up time vs 4w.
