# Supplementary material for: WeChat as a Platform for Baduanjin Intervention in Patients With Stable Chronic Obstructive Pulmonary Disease in China: Retrospective Randomized Controlled Trial
Source: JMIR Mhealth Uhealth. 2021 Feb 2;9(2):e23548. doi: 10.2196/23548 (PMC7886617; doi:10.2196/23548)
Supplement: Multimedia Appendix 2 [file mhealth_v9i2e23548_app2.docx]

**Multimedia Appendix 2.** Lung function in the WeChat group and control group.

| before Baduanjin after Baduanjin  *Z*  *P* |
| --- |
| Control group 38.65±13.68 44.1±13.63 -3.686 ＜.001  WeChat group 39.55±15.13 51.07±14.43 -6.985 ＜.001  *Z* -0.486 -3.679 — —  *P* 0.627 <0.001 — — |

Lung function，FEV1%pred.
