# Supplementary material for: WeChat as a Platform for Baduanjin Intervention in Patients With Stable Chronic Obstructive Pulmonary Disease in China: Retrospective Randomized Controlled Trial
Source: JMIR Mhealth Uhealth. 2021 Feb 2;9(2):e23548. doi: 10.2196/23548 (PMC7886617; doi:10.2196/23548)
Supplement: Multimedia Appendix 3 [file mhealth_v9i2e23548_app3.docx]

**Multimedia Appendix 3.** Chronic obstructive pulmonary disease assessment test score in the WeChat group and control group.

| before Baduanjin after Baduanjin *Z*   *P* |
| --- |
| Control group 23±6 21±5.75 -4.937 ＜.001  WeChat group 24±7 18±6 -5.246 ＜.001  *Z* -1.407 -5.246 — —  *P* 0.295 ＜0.001 — — |

CAT，COPD assessment test.
