# Supplementary material for: WeChat as a Platform for Baduanjin Intervention in Patients With Stable Chronic Obstructive Pulmonary Disease in China: Retrospective Randomized Controlled Trial
Source: JMIR Mhealth Uhealth. 2021 Feb 2;9(2):e23548. doi: 10.2196/23548 (PMC7886617; doi:10.2196/23548)
Supplement: Multimedia Appendix 4 [file mhealth_v9i2e23548_app4.pdf]

CONSORT-EHEALTH Checklist V1.6.2 Report	Manuscript Number	23548
**(based on CONSORT-EHEALTH V1.6), available at [http://tinyurl.com/consort-ehealth-v1-6].**		
**Date completed**		
1/25/2021 21:51:02		
**by**		
Junjie Bi		
WeChat as a Platform for Baduanjin Intervention in Patients WithStable Chronic Obstructive Pulmonary Disease in China:Retrospective Randomized Controlled Trial		
TITLE		
**1a-i) Identify the mode of delivery in the title**		
WeChat is an open, widely used app.		
**1a-ii) Non-web-based components or important co-interventions in title**		
**1a-iii) Primary condition or target group in the title**		
WeChat as a Platform for Baduanjin Intervention in Patients With "Stable Chronic Obstructive Pulmonary Disease" in China:Retrospective Randomized Controlled Trial		
ABSTRACT		
**1b-i) Key features/functionalities/components of the intervention and comparator in the METHODS section of the ABSTRACT**		
For further information see manuscript, heading "HEADING".		
**1b-ii) Level of human involvement in the METHODS section of the ABSTRACT**		
**1b-iii) Open vs. closed, web-based (self-assessment) vs. face-to-face assessments in the METHODS section of the ABSTRACT**		
**1b-iv) RESULTS section in abstract must contain use data**		
**1b-v) CONCLUSIONS/DISCUSSION in abstract for negative trials**		
INTRODUCTION		
**2a-i) Problem and the type of system/solution**		
For further information see manuscript, heading "HEADING".		
**2a-ii) Scientific background, rationale: What is known about the (type of) system**		
For further information see manuscript, heading "HEADING".		
**Does your paper address CONSORT subitem 2b?**		
For further information see manuscript, heading "HEADING".		
METHODS		
**3a) CONSORT: Description of trial design (such as parallel, factorial) including allocation ratio**		
This study was a "parallel controlled study" conducted from September 2018 to October 2019 in Shanghai, China. Two hundred stable COPD patients were included, and the participants were randomly divided into the following two groups "(1:1 ratio)": the WeChat intervention group and routine nursing control group (Figure 1).		
**3b) CONSORT: Important changes to methods after trial commencement (such as eligibility criteria), with reasons**		
No.		
**3b-i) Bug fixes, Downtimes, Content Changes**		
**4a) CONSORT: Eligibility criteria for participants**		
"The inclusion criteria were as follows: (1) 50 to 80 years of age regardless of gender; (2) confirmed clinical diagnosis of stable COPD according to the standard of GOLD 2018 [20]; (3) informed consent (patients and families); (4) ability to use WeChat proficiently (patients and primary caregivers); and (5) ability to perform Baduanjin independently.<br><br>The exclusion criteria were as follows: (1) severe heart, liver, and kidney diseases, tumors, or other conditions that may affect the observation; (2) life expectancy less than 1 year; and (3) history of conducting physical exercise for a long time (≥3 times/week, ≥20 minutes/time, persisting for more than 12 months) [21]."		
**4a-i) Computer / Internet literacy**		
**4a-ii) Open vs. closed, web-based vs. face-to-face assessments:**		
"All patients with stable COPD were discharged from the respiratory medicine ward of a large general hospital.All candidates first completed a brief screening questionnaire. Patients who met the inclusion criteria were invited to participate in the study and received more detailed information regarding the study. After providing written informed consent, all qualified patients were divided into the WeChat group and control group. "		
**4a-iii) Information giving during recruitment**		
**4b) CONSORT: Settings and locations where the data were collected**		
"The Baduanjin exercise frequency was collected each week in WeChat and once a week by telephone. At the end of the study, the lung function evaluation, quality of life evaluation (CAT), personal activity evaluation, and satisfaction survey were completed by all participants."		
**4b-i) Report if outcomes were (self-)assessed through online questionnaires**		
At the end of the study, the lung function evaluation, quality of life evaluation (CAT), personal activity evaluation, and" satisfaction survey "were completed by all participants.		
**4b-ii) Report how institutional affiliations are displayed**		
**5) CONSORT: Describe the interventions for each group with sufficient details to allow replication, including how and when they were actually administered**		
**5-i) Mention names, credential, affiliations of the developers, sponsors, and owners**		
**5-ii) Describe the history/development process**		
**5-iii) Revisions and updating**		
**5-iv) Quality assurance methods**		
**5-v) Ensure replicability by publishing the source code, and/or providing screenshots/screen-capture video, and/or providing flowcharts of the algorithms used**		
**5-vi) Digital preservation**		
**5-vii) Access**		
"WeChat is the mainstream instant communication platform in China [13]. The "2018 WeChat Annual Data Report" reported that 1.01 billion users logged on to WeChat daily in 2018. The function of the platform is quite strong but is very easy to operate."		
**5-viii) Mode of delivery, features/functionalities/components of the intervention and comparator, and the theoretical framework**		
For further information see manuscript, heading "HEADING".		
**5-ix) Describe use parameters**		
**5-x) Clarify the level of human involvement**		
**5-xi) Report any prompts/reminders used**		
WeChat is an open platform for the masses, and we have no control over its various other reminders.		
**5-xii) Describe any co-interventions (incl. training/support)**		
"The patients very actively participated in the communication. Responses to all questions were obtained from the medical staff in a timely manner."		

**6a) CONSORT: Completely defined pre-specified primary and secondary outcome measures, including how and when they were assessed**		
For further information see manuscript, heading "HEADING".		
**6a-i) Online questionnaires: describe if they were validated for online use and apply CHERRIES items to describe how the questionnaires were designed/deployed**		
**6a-ii) Describe whether and how “use” (including intensity of use/dosage) was defined/measured/monitored**		
**6a-iii) Describe whether, how, and when qualitative feedback from participants was obtained**		
**6b) CONSORT: Any changes to trial outcomes after the trial commenced, with reasons**		
"The Baduanjin exercise frequency was collected each week in WeChat and once a week by telephone. At the end of the study, the lung function evaluation, quality of life evaluation (CAT), personal activity evaluation, and satisfaction survey were completed by all participants."		
**7a) CONSORT: How sample size was determined**		
**7a-i) Describe whether and how expected attrition was taken into account when calculating the sample size**		
**7b) CONSORT: When applicable, explanation of any interim analyses and stopping guidelines**		
For further information see manuscript, heading "HEADING".		
**8a) CONSORT: Method used to generate the random allocation sequence**		
For further information see manuscript, heading "HEADING".		
**8b) CONSORT: Type of randomisation; details of any restriction (such as blocking and block size)**		
"Using the method of block randomization, 200 research patients were randomly assigned to the WeChat and control groups. The block size was defined as four, and there were six sequential arrangements and combinations. Excel (Microsoft Corp) randomly generated 50 (1–50) numbers that did not repeat. Then, the numbers were divided by six to obtain the remainder, and six combinations were matched according to the remainder. A random block group table was then completed. After the research patients were included, the random block table was assigned to a group. By the nature of the trial design, neither the research staff nor the participants were blinded to the intervention."		
**9) CONSORT: Mechanism used to implement the random allocation sequence (such as sequentially numbered containers), describing any steps taken to conceal the sequence until interventions were assigned**		
"Using the method of block randomization, 200 research patients were randomly assigned to the WeChat and control groups. The block size was defined as four, and there were six sequential arrangements and combinations. Excel (Microsoft Corp) randomly generated 50 (1–50) numbers that did not repeat. Then, the numbers were divided by six to obtain the remainder, and six combinations were matched according to the remainder. A random block group table was then completed. After the research patients were included, the random block table was assigned to a group. By the nature of the trial design, neither the research staff nor the participants were blinded to the intervention."		
**10) CONSORT: Who generated the random allocation sequence, who enrolled participants, and who assigned participants to interventions**		
"Using the method of block randomization, 200 research patients were randomly assigned to the WeChat and control groups. The block size was defined as four, and there were six sequential arrangements and combinations. Excel (Microsoft Corp) randomly generated 50 (1–50) numbers that did not repeat. Then, the numbers were divided by six to obtain the remainder, and six combinations were matched according to the remainder. A random block group table was then completed. After the research patients were included, the random block table was assigned to a group. By the nature of the trial design, neither the research staff nor the participants were blinded to the intervention."		
**11a) CONSORT: Blinding - If done, who was blinded after assignment to interventions (for example, participants, care providers, those assessing outcomes) and how**		
**11a-i) Specify who was blinded, and who wasn’t**		
"Using the method of block randomization, 200 research patients were randomly assigned to the WeChat and control groups. The block size was defined as four, and there were six sequential arrangements and combinations. Excel (Microsoft Corp) randomly generated 50 (1–50) numbers that did not repeat. Then, the numbers were divided by six to obtain the remainder, and six combinations were matched according to the remainder. A random block group table was then completed. After the research patients were included, the random block table was assigned to a group. By the nature of the trial design, neither the research staff nor the participants were blinded to the intervention."		
**11a-ii) Discuss e.g., whether participants knew which intervention was the “intervention of interest” and which one was the “comparator”**		
**11b) CONSORT: If relevant, description of the similarity of interventions**		
No.		
**12a) CONSORT: Statistical methods used to compare groups for primary and secondary outcomes**		
For further information see manuscript, heading "HEADING".		
**12a-i) Imputation techniques to deal with attrition / missing values**		
No.		
**12b) CONSORT: Methods for additional analyses, such as subgroup analyses and adjusted analyses**		
No.		
RESULTS		
**13a) CONSORT: For each group, the numbers of participants who were randomly assigned, received intended treatment, and were analysed for the primary outcome**		
For further information see manuscript, heading "HEADING".		
**13b) CONSORT: For each group, losses and exclusions after randomisation, together with reasons**		
No.		
**13b-i) Attrition diagram**		
**14a) CONSORT: Dates defining the periods of recruitment and follow-up**		
The Baduanjin exercise frequency was collected" each week in WeChat and once a week " by telephone. "At the end of the study (3 months)", the lung function evaluation, quality of life evaluation (CAT), personal activity evaluation, and satisfaction survey were completed by all participants.		
**14a-i) Indicate if critical “secular events” fell into the study period**		
**14b) CONSORT: Why the trial ended or was stopped (early)**		
No.		
**15) CONSORT: A table showing baseline demographic and clinical characteristics for each group**		
For further information see manuscript, heading "HEADING"		
**15-i) Report demographics associated with digital divide issues**		
For further information see manuscript, heading "HEADING".		
**16a) CONSORT: For each group, number of participants (denominator) included in each analysis and whether the analysis was by original assigned groups**		
**16-i) Report multiple “denominators” and provide definitions**		
For further information see manuscript, heading "HEADING".		
**16-ii) Primary analysis should be intent-to-treat**		
**17a) CONSORT: For each primary and secondary outcome, results for each group, and the estimated effect size and its precision (such as 95% confidence interval)**		
For further information see manuscript.		
**17a-i) Presentation of process outcomes such as metrics of use and intensity of use**		
**17b) CONSORT: For binary outcomes, presentation of both absolute and relative effect sizes is recommended**		
For further information see manuscript.		
**18) CONSORT: Results of any other analyses performed, including subgroup analyses and adjusted analyses, distinguishing pre-specified from exploratory**		
For further information see manuscript.		
**18-i) Subgroup analysis of comparing only users**		
**19) CONSORT: All important harms or unintended effects in each group**		
No.		
**19-i) Include privacy breaches, technical problems**		
**19-ii) Include qualitative feedback from participants or observations from staff/researchers**		

DISCUSSION		
**20) CONSORT: Trial limitations, addressing sources of potential bias, imprecision, multiplicity of analyses**		
**20-i) Typical limitations in ehealth trials**		
For further information see manuscript, heading "HEADING".		
**21) CONSORT: Generalisability (external validity, applicability) of the trial findings**		
**21-i) Generalizability to other populations**		
**21-ii) Discuss if there were elements in the RCT that would be different in a routine application setting**		
**22) CONSORT: Interpretation consistent with results, balancing benefits and harms, and considering other relevant evidence**		
**22-i) Restate study questions and summarize the answers suggested by the data, starting with primary outcomes and process outcomes (use)**		
For further information see manuscript.		
**22-ii) Highlight unanswered new questions, suggest future research**		
Other information		
**23) CONSORT: Registration number and name of trial registry**		
No. This randomized study was [only retrospectively | not] registered because of technical issues and the limitation of time.		
**24) CONSORT: Where the full trial protocol can be accessed, if available**		
The full trial protocol can be accessed in our department.		
**25) CONSORT: Sources of funding and other support (such as supply of drugs), role of funders**		
This work was supported by the National Key Research and Development Program of China (number: 2018YFC1313600), the Shanghai Science and Technology Committee (number: 18140904000, 17401970900), Department of Respiratory Medicine Development Fund of Putuo District (2016PTZK03), the Scientific Innovation Foundation of Putuo District (ptkwws201714), Shanghai Municipal Commission of Health and Family Planning (20174Y0239), the Peiying Program of Putuo Hospital (2017206A), and the scientific project of Shanghai University of Traditional Chinese Medicine (2019LK096) and Specialty Construction of Respiratory and Critical Care Medicine (2020tszk02).		
**X26-i) Comment on ethics committee approval**		
**x26-ii) Outline informed consent procedures**		
**X26-iii) Safety and security procedures**		
**X27-i) State the relation of the study team towards the system being evaluated**		
